# Cell-division pattern and phylogenetic analyses of a new ciliate genus *Parasincirra* n. g. (Protista, Ciliophora, Hypotrichia), with a report of a new soil species, *P. sinica* n. sp. from northwest China

**DOI:** 10.1186/s12862-020-01730-4

**Published:** 2021-02-10

**Authors:** Jiyang Ma, Yan Zhao, Tengyue Zhang, Chen Shao, Khaled A. S. Al-Rasheid, Weibo Song

**Affiliations:** 1 Institute of Evolution & Marine Biodiversity, and College of Fisheries, Ocean University of China, Qingdao, 266003 China; 2 Laboratory of Protozoological Biodiversity and Evolution in Wetland, College of Life Sciences, Shaanxi Normal University, Xi’an, 710119 China; 3 College of Life Sciences, Capital Normal University, Beijing, 100048 China; 4 Zoology Department, College of Science, King Saud University, Riyadh, 11451 Saudi Arabia; 5 Laboratory for Marine Biology and Biotechnology, Qingdao National Laboratory for Marine Science and Technology, Qingdao, 266003 China

**Keywords:** New species, Morphology, Morphogenesis, *Parasincirra*, SSU rDNA phylogeny

## Abstract

**Background:**

Ciliated protists, a huge assemblage of unicellular eukaryotes, are extremely diverse and play important ecological roles in most habitats where there is sufficient moisture for their survivals. Even though there is a growing recognition that these organisms are associated with many ecological or environmental processes, their biodiversity is poorly understood and many biotopes (e.g. soils in desert areas of Asia) remain largely unknown. Here we document an undescribed form found in sludge soil in a halt-desert inland of China. Investigations of its morphology, morphogenesis and molecular phylogeny indicate that it represents a new genus and new species, *Parasincirra sinica* n. g., n. sp.

**Results:**

The new, monotypic genus *Parasincirra* n. g. is defined by having three frontal cirri, an amphisiellid median cirral row about the same length as the adoral zone, one short frontoventral cirral row, cirrus III/2 and transverse cirri present, buccal and caudal cirri absent, one right and one left marginal row and three dorsal kineties. The main morphogenetic features of the new taxon are: (1) frontoventral-transverse cirral anlagen II to VI are formed in a primary mode; (2) the amphisiellid median cirral row is formed by anlagen V and VI, while the frontoventral row is generated from anlage IV; (3) cirral streaks IV to VI generate one transverse cirrus each; (4) frontoventral-transverse cirral anlage II generates one or two cirri, although the posterior one (when formed) will be absorbed in late stages, that is, no buccal cirrus is formed; (5) the posterior part of the parental adoral zone of membranelles is renewed; (6) dorsal morphogenesis follows a typical *Gonostomum*-pattern; and (7) the macronuclear nodules fuse to form a single mass. The investigation of its molecular phylogeny inferred from Bayesian inference and Maximum likelihood analyses based on small subunit ribosomal DNA (SSU rDNA) sequence data, failed to reveal its exact systematic position, although species of related genera are generally assigned to the family Amphisiellidae Jankowski, 1979*.* Morphological and morphogenetic differences between the new taxon and *Uroleptoides* Wenzel, 1953, *Parabistichella* Jiang et al., 2013, and other amphisiellids clearly support the validity of *Parasincirra* as a new genus. The monophyly of the family Amphisiellidae is rejected by the AU test in this study.

**Conclusions:**

The critical character of the family Amphisiellidae, i.e., the amphisiellid median cirral row, might result from convergent evolution in different taxa. Amphisiellidae are not monophyletic.

## Background

Recent faunistic studies have revealed numerous new taxa of hypotrichous ciliates suggesting that this group is more diverse than previously supposed [1–8]. Furthermore, much work has been carried out on the morphogenesis and molecular phylogeny of hypotrichs, which has led to a better understanding of their systematics and evolutionary relationships [9–17].

**Fig. 1:**
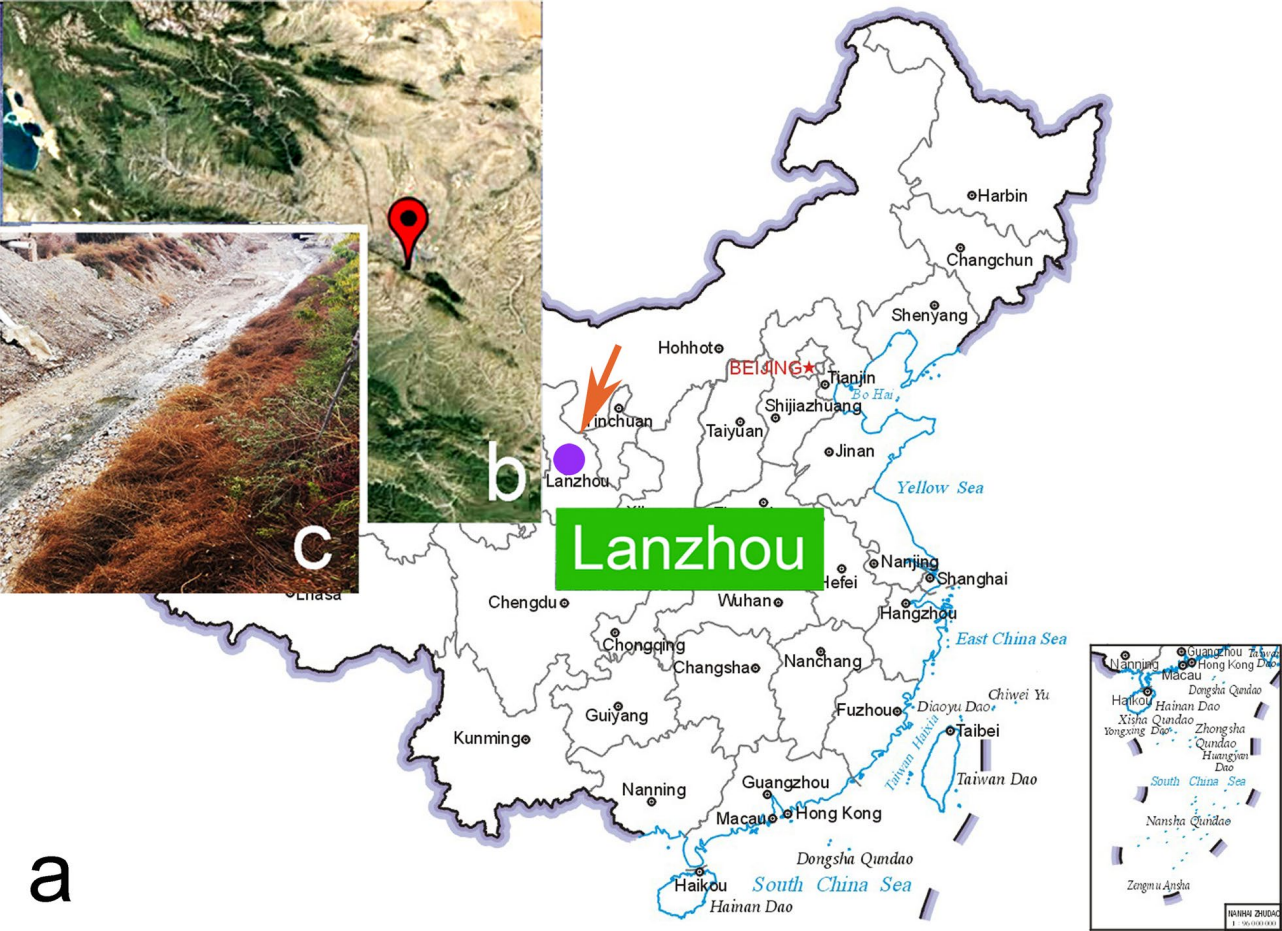
**a**–**c** Locations of the sample sites. **a**, **b** The map of China from the MAP WORLD (www.tianditu.gov.cn, drawing review number: GS (2019) 1673) (a) and portion of Google Map (b), showing the location of Lanzhou, China (36º 03′ N; 103º 49′ E). **c** Showing the area surrounding the flood drain from where the sample containing *Parasincirra sinica* n. sp. was collected

Among these, the order Stichotrichida Fauré-Fremiet, 1961 is one of the most confused and diverse ciliate groups in terms of both its taxonomy and phylogeny [18]. One of its largest families, Amphisiellidae Jankowski, 1979, is characterised by the possession of an amphisiellid median cirral row derived from two or three, rather than one, frontoventral-transverse cirral anlagen. Most amphisiellids occur in terrestrial habitats, although some are marine [15, 19, 20]. In the present study, we present a new amphisiellid collected from sludge soil in a flood drain in Lanzhou, China (Fig. 1). Observations of its morphology and morphogenesis, both *in vivo* and after protargol staining, demonstrate that it represents a novel genus, *Parasincirra* n. g., of the family Amphisiellidae. The SSU rDNA of the new isolate was sequenced and its molecular phylogeny was analyzed.

## Results

### ZooBank registration

Present work: urn:lsid:zoobank.org:pub:51385DEC-9698-435A-AB33-C3EB91CBE777.

### Establishment of the new genus *Parasincirra* n. g.

#### ZooBank registration

*Parasincirra* n. g.: urn:lsid:zoobank.org:act:ACDEF2AA-F1A6-4D80-B0F9-115603AB6B3F.

*Diagnosis* Amphisiellidae with elongate body. Three frontal cirri. Amphisiellid median cirral row about same length as adoral zone. One short frontoventral cirral row. Cirrus III/2 and transverse cirri present. One right and one left marginal row. Three dorsal kineties. Caudal cirri and buccal cirrus lacking.

*Type species* *Parasincirra sinica* n. sp.

*Etymology* Composite of the Greek prefix *para-* (close to; related; deviating) and suffix (*-sincirra*) of the genus name *Hemisincirra* Hemberger, 1985. This indicates that *Parasincirra* has a cirral pattern similar to that of *Hemisincirra*. Feminine gender.

*Remarks* We do not assign *Hemisincirra interrupta* (Foissner, 1982) Foissner in Berger, 2001 and *H. vermicularis* Hemberger, 1985 to our new genus *Parasincirra* although both species also lack a buccal cirrus. The main reasons are that either the ontogenetic or the molecular information are unknown for these two species.

### *Parasincirra sinica* n. sp.

#### ZooBank registration

*Parasincirra sinica* n. sp.: urn:lsid:zoobank.org:act:3F05A977-F059-4108-895F-CA98FF59E8DE.

*Diagnosis* Size *in vivo* 90–160 μm × 20–40 μm. Body slender, fusiform to vermiform, with pointed posterior end. Two to six (mostly four) macronuclear nodules. Contractile vacuole located slightly ahead of mid-body. Cortical granules about 0.5 μm across, colourless and grouped around dorsal ciliary organelles. Three frontal cirri and one parabuccal cirrus; frontoventral row constantly with two cirri; two to four transverse cirri. Amphisiellid median cirral row terminates behind level of cytostome, invariably composed of four cirri. One left and one right marginal row, composed of 34–52 and 34–53 cirri respectively. Three bipolar dorsal kineties. Adoral zone composed of 14–19 membranelles. Soil habitat.

*Type material* One protargol-stained slide (no. MJY2017043001B) with the holotype specimen and two paratype slides (no. MJY2017043001A, C) were deposited in the Laboratory of Protozoological Biodiversity and Evolution in Wetland, Shaanxi Normal University, China.

*Type locality* Flood drain, Lanzhou (36º 03′ N; 103º 49′ E), China.

*Etymology* The species-group name *sinica* means the species was first discovered in China.

**Fig. 2:**
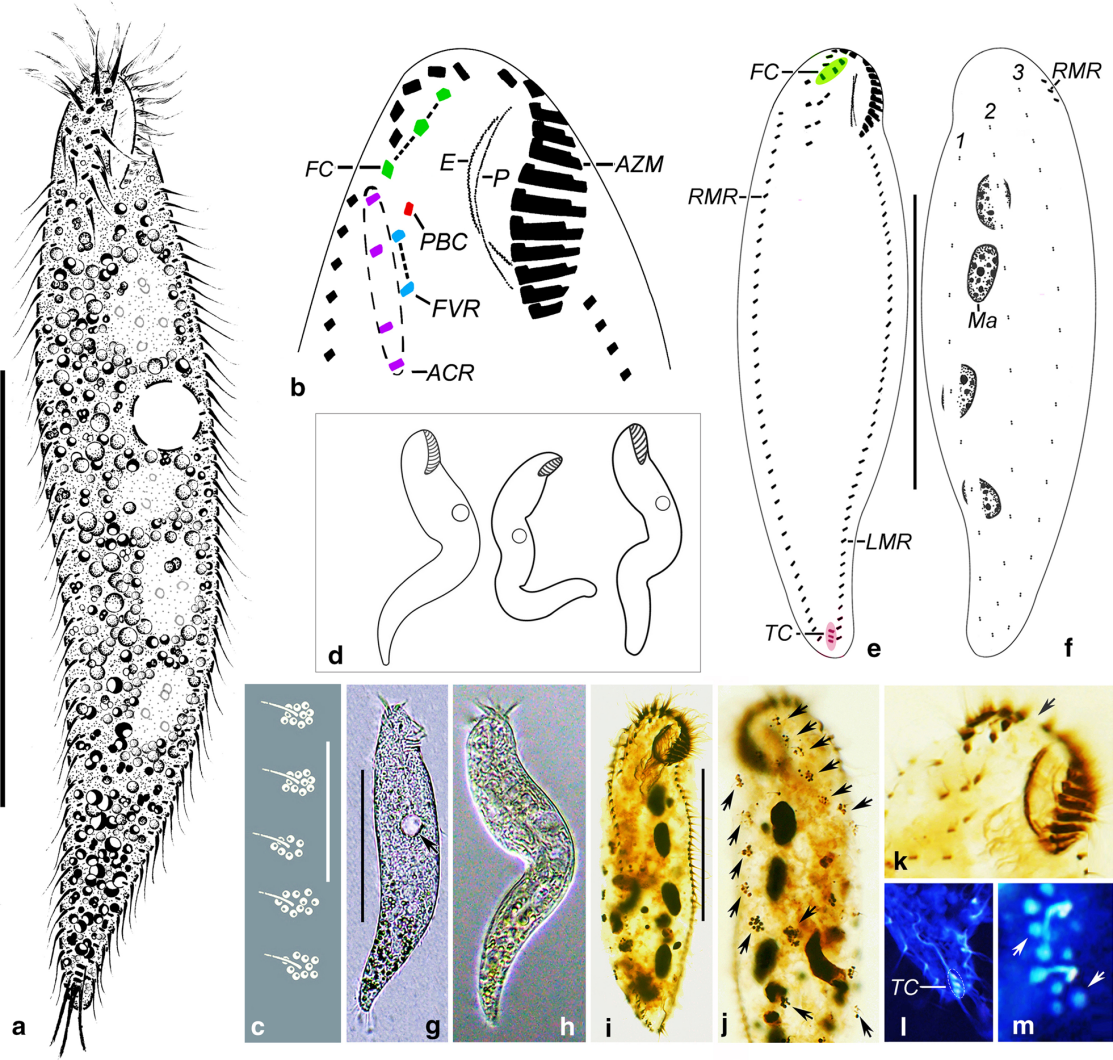
**a**–**m** Morphology of *Parasincirra sinica* n. sp. from life (a, c, d, g, h) and after protargol staining (b, e, f, i–m). **a** Ventral view of a representative specimen. **b** Ventral view, to show ciliature of frontoventral area. **c** Arrangement of cortical granules on dorsal side. **d** Ventral views, to show the various body shapes. **e**, **f** Ventral (e) and dorsal (f) view of a typical individual, to show the ciliature and nuclear apparatus. **g**, **h** Ventral views of representative individuals, arrow indicates contractile vacuole. **i** Ventral view of the holotype specimen to show ventral ciliature. **j** Dorsal view to show cortical granules (arrows). **k** Ventral view of anterior portion, to show the cirri in frontoventral area and a short gap in adoral zone of membranelles (arrow). **l** Ventral view, to show transverse cirri. **m**. Showing cortical granules on dorsal body side (arrows). *ACR* amphisiellid cirral row, *AZM* adoral zone of membranelles, *E* endoral, *FC* frontal cirri, *FVR* frontoventral cirral row, *LMR* left marginal row, *Ma* macronuclear nodules, *Mi* micronuclei, *P* paroral, *PBC* parabuccal cirri, *RMR* right marginal row, *TC* transverse cirri, *1–3* dorsal kineties 1–3. Scale bars: **a**, **e**, **f**, **i** = 60 μm, **c** = 15 μm. The images of this figure we have used are freely available to use

*Morphological description* Body 90–160 μm × 20–40 μm *in vivo* (n = 6) with a ratio of length to width of about 3.5:1–7.5:1; protargol-stained cells 120 μm × 30 μm on average with a ratio of length to width of about 4:1. Generally slender, almost fusiform to vermiform, non-contractile but highly flexible, and thus cell outline variable, i.e., sigmoidal or curved (Fig. 2d). Anterior end narrowly rounded and posterior end more or less tapered to form a pointed tail that is more flexible and contractile than the rest of the cell (Fig. 2a, d, g, h); tail unrecognisable in protargol preparations (Fig. 2e, f, i). Dorsoventrally flattened up to 2:1. Usually four (2–6) macronuclear nodules arranged along or slightly left of mid-line, behind buccal vertex; one to three, on average two, micronuclei attached, or near to, macronuclear nodules. Macronuclear nodules ellipsoidal, about 9–19 μm × 4–10 μm after protargol staining (Fig. 2j). Micronuclei about 2.9 μm × 2.4 μm after protargol staining. One contractile vacuole measuring about 13 μm in diameter in diastole, positioned near left margin, contracting at intervals of 10 s (Fig. 2g, h). Cortical granules colourless, globular, about 0.5 μm in diameter, distributed around dorsal ciliary organelles, also visible in protargol preparations (Fig. 2c, j, m). Cytoplasm colourless to greyish, often packed with numerous small lipid droplets. Locomotion mainly by slowly crawling on substrate and debris, sometimes jerking back and forth. When suspended, cells often swim continuously in circles.

Infraciliature as shown in Fig. 2b, e, f, i–l. Most somatic cirri relatively fine with cilia about 12–16 μm long. Constantly three relatively stout frontal cirri in an almost transverse pseudo-row immediately behind several distal adoral membranelles, cilia about 15 μm long. Amphisiellid median cirral row (ACR) short and consisting of four cirri; commences at about the level of the rightmost frontal cirrus (about 6% down length of body), or slightly lower, terminates at about level of buccal vertex (about 21% down length of body). Parabuccal cirrus (cirrus III/2) located at the level of the middle region of the paroral and endoral. Frontoventral row lies between the parabuccal cirrus and the ACR, invariably composed of two cirri; commences at about the level of the second cirrus in the ACR (about 8% down length of body) and terminates ahead of the third cirrus in the ACR (about 10% down length of body). Three, rarely two or four, slightly subterminal transverse cirri, cilia of which are about 16 μm long. Constantly one left and one right marginal row with 34–52 and 34–53 cirri, respectively (Table 1). Right marginal cirral row begins dorsolaterally at anterior end of cell while left marginal cirral row begins at level of posterior end of adoral zone, both terminate caudally, not confluent posteriorly (Fig. 2b, e, i, k, l).

**Table 1:** Morphometric characteristics of *Parasincirra sinica* n. sp

Character^a^	HT	Min	Max	Mean	M	SD	CV	n
Body, length	105	81	152	119.7	115	18.3	15.3	25
Body, width	29	18	47	30.3	31	6.3	21.0	25
Body, length: width ratio	3.57	2.29	7.59	4.12	3.79	1.13	27.35	25
AZM, length	16	13	24	19.1	19	2.5	13.0	25
AZM, length: body length ratio	0.16	0.11	0.20	0.16	0.16	0.02	13.07	25
AZM, number	15	14	19	15.6	15	1.3	8.1	25
Paroral, length	15	8	16	12.4	12	2.4	19.3	15
Endoral, length	13	8	15	11.0	11	2.1	18.8	15
PBC, number	1	1	1	1.0	1	0	0	25
FVR, number	2	2	2	2.0	2	0	0	25
ACR, cirri number	4	4	4	4.0	4	0	0	25
Frontal cirri, number	3	3	3	3.0	3	0	0	25
Left marginal cirri, number	43	34	52	41.3	41	5.3	12.9	25
Right marginal cirri, number	39	34	53	41.2	39	5.2	12.6	25
Transverse cirri, number	3	2	4	3.1	3	0.5	16.0	25
Dorsal kineties, number	3	3	3	3.0	3	0	0	25
Dikinetids in DK1, number	10	10	17	12.5	12	2.3	18.7	15
Dikinetids in DK2, number	15	13	18	14.9	15	1.5	10.3	15
Dikinetids in DK3, number	16	11	18	14.5	14	2.2	15.0	15
Macronuclear nodules, number	4	2	6	4.1	4	0.7	16.2	25
Macronuclear nodule, average length	10	9	19	13.8	14	2.8	20.4	25
Macronuclear nodule, average width	5	4	10	6.0	6	1.2	20.4	25
Micronuclei, number	2	1	3	2.0	2	0.7	37.5	25
Micronuclear nodule, average length	4	2	4	2.9	3	0.4	14.5	25
Micronuclear nodule, average width	3	2	4	2.4	2	0.4	18.0	25

*ACR* amphisiellid median cirral row, *AZM* adoral zone of membranelles, *CV* coefficient of variation in %, *DK* dorsal kineties, *FVR* frontoventral cirral row, *HT* holotype specimen, *M* median, *Max* maximum, *Mean* arithmetic mean, *Min* minimum, *n* sample size, *PBC* parabuccal cirri, *SD* standard deviation

^a^All data are based on protargol-stained specimens. Measurements in µm

Three dorsal kineties arranged in *Gonostomum*-pattern, with cilia about 3 μm in length, composed of about 13, 15 and 15 dikinetids, respectively, and arranged in a gradient; that is, kinety 3 commences apically, kinety 2 starts slightly behind kinety 3, while kinety 1 starts slightly behind kinety 2. Each terminates at the posterior end of the body (Fig. 2f, j).

Adoral zone of membranelles (AZM) shaped as in other amphisiellid species, terminates 11–20% (average about 16%) down length of body, comprising 14–19 membranelles. Cilia of distal membranelles about 13 μm long. Buccal cavity small, endoral and paroral bending strongly and optically intersecting with each other at their lower or middle regions (Fig. 2b, e, i, k).

### Morphogenesis during binary fission

#### Stomatogenesis

**Fig. 3:**
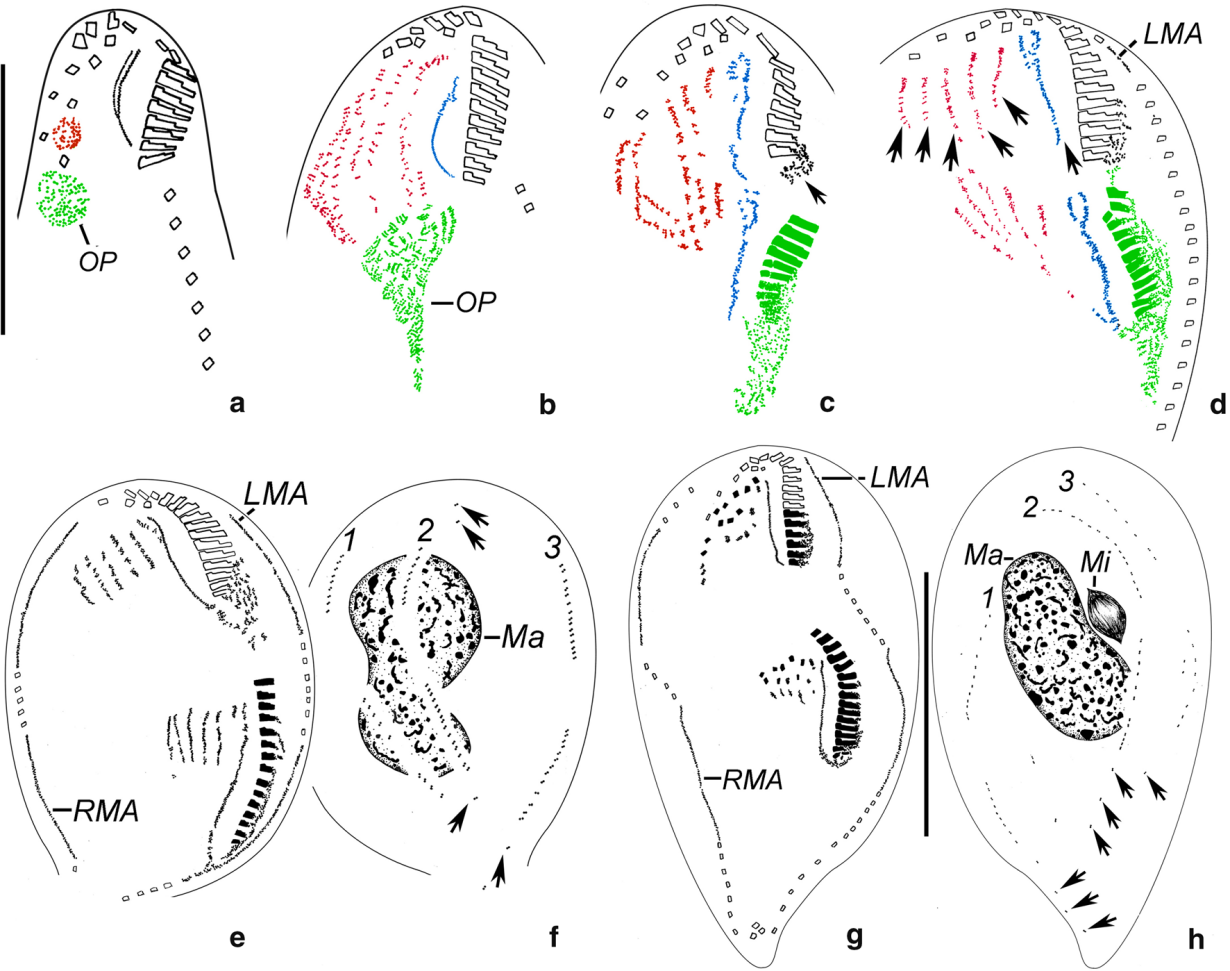
**a**–**h** Early and middle stages of morphogenesis in *Parasincirra sinica* n. sp. after protargol staining. **a**, **b** Ventral views of early dividers, showing oral primordium of opisthe and frontoventral-transverse cirral anlagen. Note parental undulating membranes start to dedifferentiate (b). **c**, **d** Ventral views of later dividers, to show the development of oral primordium, frontoventral-transverse cirral anlagen and undulating membranes anlagen (arrows). Note the dedifferentiation of membranelles at the proximal end of the old adoral zone of membranelles (arrow in c), and the intrakinetally formed anlagen for the marginal rows (d). **e**–**h** Ventral (e, g) and dorsal (f, h) views of middle dividers, to show stretched marginal anlagen and dorsal kineties anlagen, the posterior membranelles of the parental adoral zone of membranelles renewed (g) and the macronuclear nodules fusing into a single mass. Note the old dorsal dikinetids are not absorbed (arrows). *OP* oral primordium, *LMA* left marginal anlagen, *Ma* macronuclear nodules, *Mi* micronuclei, *RMA* right marginal anlagen, *1–3* dorsal kineties anlagen 1–3. Scale bars: a, g, h = 60 μm. The images of this figure we have used are freely available to use

**Fig. 4:**
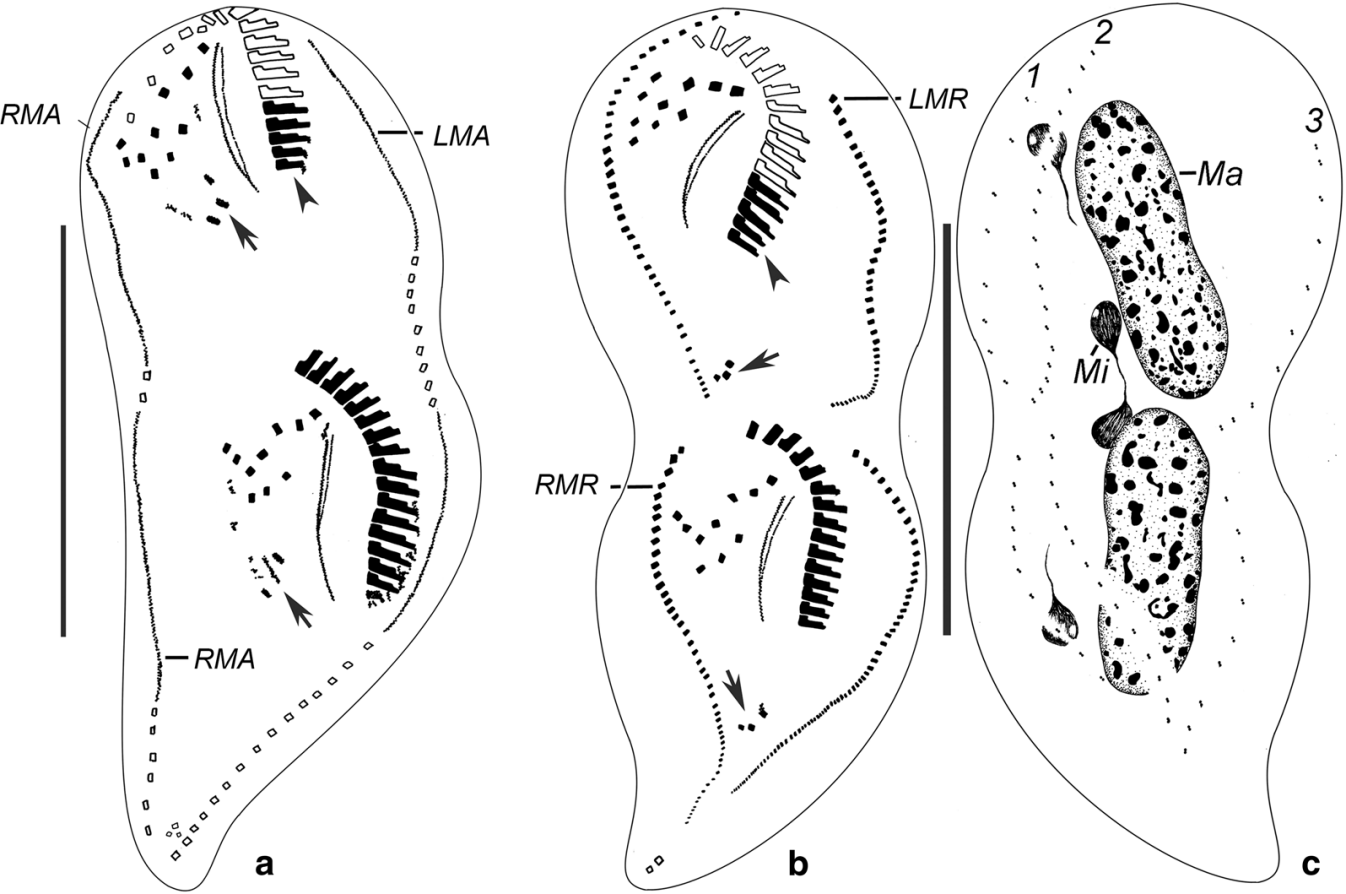
**a**–**c** Late stages of morphogenesis in *Parasincirra sinica* n. sp. after protargol staining. Ventral (a, b) and dorsal (c) views, to show the frontoventral-transverse cirral anlagen differentiating into cirri, transverse cirri migrating into their final position (arrows), the old adoral zone of membranelles have been rebuilt (arrowheads). *LMA* left marginal anlagen, *LMR* left marginal row, *RMA* right marginal anlagen, *RMR* right marginal row, *Ma* macronuclear nodules, *Mi* micronuclei, *1–3* dorsal kineties anlagen 1–3. Scale bars: 60 μm. The images of this figure we have used are freely available to use

**Fig. 5:**
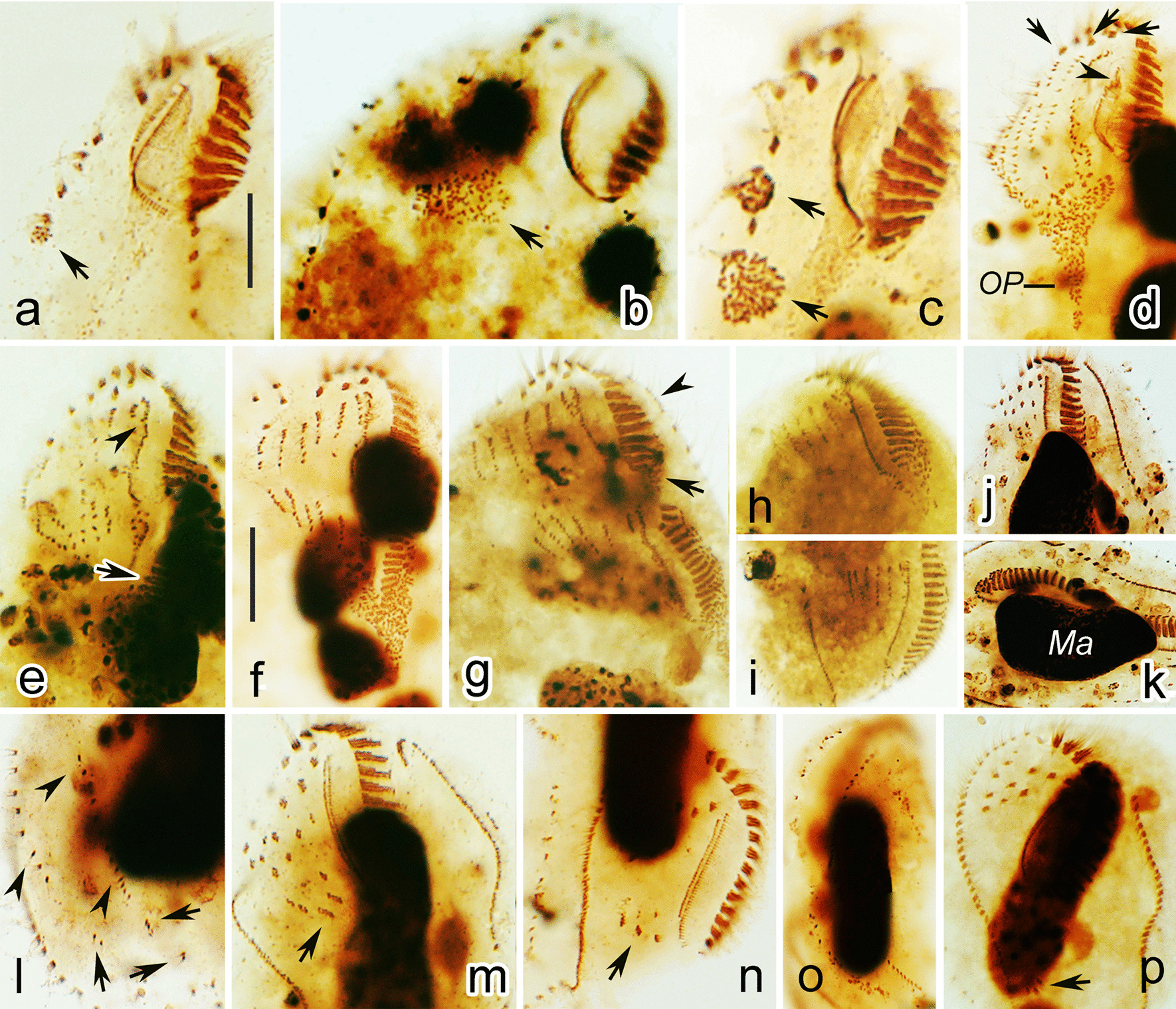
**a**–**p** Photomicrographs of *Parasincirra sinica* n. sp. during morphogenesis (after protargol staining). **a**–**d** Ventral views of early dividers, to indicate the oral primordium (arrows in a–c), the formation of frontoventral-transverse cirral anlagen and undulating membranes starting to dedifferentiate (arrowhead). Note the old frontal cirri remain intact (arrows in d). **e**, **f** Ventral views of later dividers, to show the oral primordia starting to differentiate into membranelles (arrow), formation of undulating membranes anlagen in the proter (arrowhead), and frontoventral-transverse cirral anlagen starting to separate (f). **g**–**i** Ventral views of later dividers, to show the dedifferentiation of membranelles at the proximal end of the old adoral zone of membranelles (arrow), the intrakinetally formed anlagen for the marginal rows (arrowhead), and stretched marginal anlagen and frontoventral-transverse cirral anlagen (h, i). **j**–**l** Ventral (j) and dorsal (k, l) views of a middle divider, to show frontoventral-transverse cirral anlagen differentiating into cirri (j), dorsal kineties anlagen (arrowheads), the old dorsal dikinetids (arrows) and the macronuclear nodules fusing into a single mass (k). **m**, **n** Ventral views of a late divider, arrows show transverse cirri migrating into their final positions in the opisthe (m) and proter (n). Note the undulating membranes anlagen longitudinally splitting into parorals and endorals. **o** Dorsal view, to show the newly formed dorsal kineties. **p** Ventral view, to demonstrate transverse cirri (arrow) migrating into their final positions. Scale bars: 15 μm

Cortical morphogenesis in *Parasincirra sinica* n. sp. mainly occurs in two zones: an anterior field for the proter and a posterior field for the opisthe. In the opisthe, the first evidence of stomatogenesis during cell division is the appearance of groups of basal bodies on the cell surface, i.e., the opisthe’s oral primordium, which is located in the end of the ACR, indicating that parental basal bodies are incorporated in the primordium (Fig. 3a). These groups subsequently merge by further proliferation of basal bodies forming a single anarchic field. Subsequently the new adoral membranelles organise posteriad (Figs. 3c, 5e). The anlage for the undulating membranes (anlage I) is formed to the right of the oral primordium (Figs. 3c, 5e). Later, the left frontal cirrus develops from the anterior end of the UM-anlage (Figs. 4g, 5j). During the later stages, the differentiation of membranelles is completed forming the new oral structure for the opisthe. Subsequently, the UM-anlage gives rise to the leftmost frontal cirrus and the new endoral and paroral (Figs. 4a, b, 5n).

In the proter, several of the proximal membranelles dedifferentiate into sparsely distributed basal bodies which then differentiate into membranelles (Fig. 3c–e). The parental undulating membranes dedifferentiate into UM-anlage. The basic development of the UM-anlage then follows a similar pattern to that in the opisthe (Figs. 3b–e, g, 4a, 5d, e, m).

#### Development of the frontoventral-transverse cirri

The development of the somatic ciliature begins with the formation of the frontoventral-transverse cirral anlagen (FVT-anlagen). Initially, the FVT-anlagen appear as a small group of basal bodies (Fig. 3a). The parental frontoventral cirri disaggregate and appear to in the formation of the FVT-anlagen. Later, five FVT-anlagen are formed to the right of the UM-anlage in the proter as primary primordia (Figs. 3b, 5d). Then, the FVT-anlagen fragment in the middle to form two sets of anlagen, one set for the proter and the other for the opisthe (Figs. 3d, e, 5h, i). Subsequently, cirri segregate from anterior to posterior in the following manner: anlage I develops the frontal cirrus I/1 (leftmost frontal cirrus); anlage II produces the middle frontal cirrus; anlage III generates a parabuccal cirrus and the rightmost frontal cirrus; anlage IV contributes two cirri forming the short frontoventral cirral row; anlage V produces the posterior two cirri in the ACR; anlage VI forms the anterior two cirri in the ACR; and anlagen IV–VI produce one transverse cirrus each (Figs. 4a, b, 5m, p). Finally, the new cirri move to their final positions.

#### Development of marginal rows and dorsal kineties

Within every parental marginal row a few cirri near the anterior end, and a few others below the mid-body, differentiate to form two separate anlagen. The dorsal kineties develop by intrakinetal basal body proliferation, i.e. two anlagen develop in each parental row. Subsequently, the new marginal cirral rows and dorsal kineties develop and replace the old ones (Figs. 3d–h, 4a–c, 5g, h, j, m).

#### Division of nuclear apparatus

The nuclear apparatus divides in the usual way for hypotrichs hence no need to describe this process in detail (Figs. 3f, h, 4c, 5k).

### SSU rDNA gene sequence and phylogenetic analyses

The 18S rDNA gene sequence of *Parasincirra sinica* n. sp. (GenBank accession number: MN472864) is 1731 bp long and has a G + C content of 45.70%. Phylogenetic trees inferred from the SSU rDNA sequences using two different methods (ML and BI) show similar topologies. Therefore, only the topology of the ML tree is presented with nodal support from both methods (Fig. 7).

Molecular phylogenetic analyses result in a clade containing four polytomies represented by *Parasincirra sinica* n. sp., two *Uroleptoides* species and *Parabistichella variabilis* Jiang et al., 2013 with high support (83% ML, 1.00 BI, Fig. 7). They also confirm the polyphyly of other amphisiellids including species belonging to the type genus *Amphisiella* Gourret and Roeser, 1888. The monophyly of the family Amphisiellidae is rejected by the AU test (p < 0.05) based on SSU-rDNA dataset (Table 2).

**Table 2:** Approximately unbiased (AU) test results

Datasets	Topology constraints	−lnL (likelihood)	AU (*P*)	Conclusion
SSU-rDNA	**Unconstrained**	8986.1376	1.000	–
	Amphisiellidae^a^	9053.5857	5e−005	**Rejected**

Significant differences (*P *value < 0.05) between the best maximum likelihood trees and the best constrained topologies are shown in bold

^a^Amphisiellidae: includes *Uroleptoides*, *Parasincirra*, *Lamtostyla* and *Amphisiella*

## Discussion

### Comparison with similar genera

Amphisiellidae were divided into three groups by Berger (2008) [19]. Group I comprises the marine taxa *Amphisiella*, *Caudiamphisiella* Berger, 2008, *Maregastrostyla* Berger, 2008 and *Spiroamphisiella* Li et al., 2007. Species of these four genera possess a buccal cirrus and a very prominent ACR which commences at about the level of the distal end of the adoral zone of membranelles and terminates beyond the mid-body. Hence the new genus, *Parasincirra* n. g., can be distinguished from the members of group I.

Group II comprises two genera, i.e., *Lamtostyla* Buitkamp, 1977 and *Uroleptoides* Wenzel, 1953, both of which possess a buccal cirrus whereas *Parasincirra* n. g. lacks a buccal cirrus.

Group III also comprises two genera, i.e. *Lamtostylides* Berger, 2008 and *Paramphisiella* Foissner, 1988. Species of these genera possess a buccal cirrus and have only one cirrus (cirrus III/2) left of the ACR. In contrast, *Parasincirra* n. g. has no buccal cirrus and one frontoventral cirrus left of the ACR.

Six genera, namely *Afroamphisiella* Foissner et al., 2002, *Cossothigma* Jankowski, 1978, *Hemisincirra* Hemberger, 1985, *Mucotrichidium* Foissner et al., 1990, *Terricirra* Berger & Foissner, 1989 and *Tetrastyla* Schewiakoff, 1892, are *incertae sedis* in Amphisiellidae [19]. With reference to the general infraciliature, *Hemisincirra* resembles *Parasincirra* n. g., however, the type species of *Hemisincirra* has a buccal cirrus (vs. absent in *Parasincirra* n. g.) [19]. *Afroamphisiella* can be distinguished from *Parasincirra* n. g. by the presence (vs. absence) of a buccal cirrus and the absence (vs. presence) of transverse cirri [19]. *Cossothigma* can be separated from the new genus by its trachelostylid (vs. elliptical to elongate-elliptical) body shape and trachelostylid oral apparatus (vs. in *Oxytricha*-pattern), and the probable presence (vs absence) of caudal cirri [19]. *Mucotrichidium* differs from the new genus in possessing a buccal cirrus, postperistomial cirrus and caudal cirri, all of which are absent in *Parasincirra* n. g. [19]. *Terricirra* can be separated from *Parasincirra* n. g. by the presence (vs. absence) of a buccal cirrus, while *Tetrastyla* can be separated from *Parasincirra* n. g. by the absence (vs. presence) of parabuccal cirri [19].

### Comparison of *Parasincirra sinica* n. sp. with similar species

Species assigned to *Hemisincirra* have an infraciliature which is very similar to that of *Parasincirra sinica* n. sp., i.e., three frontal cirri, a short amphisiellid median cirral row, few transverse cirri and lack of caudal cirri.

Considering its somatic ciliature, *Parasincirra sinica* n. sp. resembles *Hemisincirra interrupta* and *H. vermicularis* most in that these two species also lack buccal cirrus. Nevertheless *H. interrupta* can be separated from *P. sinica* n. sp. by in having fewer dorsal kineties (1 vs. 3), more macronuclear nodules (about 30 vs. 2–6) and more cirri in the amphisiellid median cirral row (6–8 vs. invariably 4). *Hemisincirra vermicularis* differs from *P. sinica* n. sp. in having more macronuclear nodules (about 10 vs. 2–6) and contractile vacuoles (4 vs. 1), and fewer dorsal kineties (1 vs. 3) [19].

In terms of the somatic ciliature, *Lamtostyla decorata* Foissner et al., 2002, *L. perisincirra* (Hemberger, 1985) Berger and Foissner, 1987, *L. islandica* Berger and Foissner, 1988, *Uroleptoides magnigranulosus* (Foissner, 1988) Berger, 2008 and *U. longiseries* (Foissner et al., 2002) Berger, 2008 closely resemble *P. sinica *n. sp. and thus should be compared to the latter. *Parasincirra sinica* n. sp. differs from *Lamtostyla decorata* in: (i) its smaller body size *in vivo* (90–160 μm × 20–40 μm vs. 100–220 μm × 20–35 μm); (ii) buccal cirrus and pretransverse cirri absent (vs. present); and (iii) fewer transverse cirri (two to four vs. five to nine) [19].

Discrepancies between *Parasincirra sinica* n. sp. and *Lamtostyla perisincirra* include: (i) its larger body size *in vivo* (90–160 μm × 20–40 μm vs. 50–80 μm × 20–30 μm); (ii) cell outline fusiform (vs. parallel body margins with both ends broadly rounded); (iii) buccal cirrus absent (vs. present); (iv) larger number of cirri in ACR (four vs. six to eight); and (v) cortical granules present (vs. absent) [19].

*Parasincirra sinica* n. sp. appears to be a close form to *Lamtostyla islandica*, but the former can be recognised by: (i) larger body size *in vivo* (90–160 μm × 20–40 μm vs. 60–80 μm × 20–25 μm); (ii) cell outline fusiform (vs. parallel body margins with both ends broadly rounded); (iii) buccal cirrus absent (vs. present); (iv) cortical granules present (vs. absent); and (v) arrangement of endoral and paroral (at about same level vs. overlapping only by about half of their length) [19].

*Uroleptoides magnigranulosus* has a close relationship to *P. sinica* n. sp. in the SSU rDNA tree (Fig. 7). *Parasincirra sinica* n. sp., however, can be recognised by: (i) buccal cirrus absent (vs. present) and (ii) having fewer cirri in the ACR (4 vs. 12–19) and transverse cirri (two to four vs. constantly five) [19].

*Parasincirra sinica* n. sp. can be separated from *Uroleptoides longiseries* by its lack of a buccal cirrus (vs. present in the latter) and having fewer cirri in the ACR (4 vs. 24–54 in the latter) [19].

### Morphogenetic comparison

One of the most remarkable morphogenetic features in *Parasincirra sinica* n. sp. is that the rightmost frontoventral row is formed by two anlagen, which is a specific character for amphisiellids and is called the amphisiellid median cirral row. Hitherto, accounts of morphogenesis are available for relatively few amphisiellids and include a wide diversity of processes:

1. The parental adoral zone of membranelles is completely retained in some taxa, e.g. *Amphisiella, Lamtostyla, Lamtostylides*, *Paramphisiella* and *Hemisincirra inquieta* Hemberger, 1985, while it is partly renewed in others, e.g. *Parasincirra* n. g*.*;
2. Ventral cirri develop from five (e.g. *Lamtostylides* and *Paramphisiella*), six (e.g. *Amphisiella*, *Parasincirra* n. g., *Spiroamphisiella*, *Hemisincirra inquieta*, *Terricirra*, *Mucotrichidium* and most *Lamtostyla* species) or seven (e.g. *Lamtostyla salina* Dong, et al., 2016) FVT-anlagen;
3. FVT-anlage II generates the buccal cirrus in several taxa (*Amphisiella*, *Spiroamphisiella*, *Lamtostyla*, *Lamtostylides*, *Paramphisiella*, *Afroamphisiella, Hemisincirra inquieta*, *Terricirra* and *Mucotrichidium*) but not in *Parasincirra* n. g.;
4. The amphisiellid median cirral row is formed by two (in *Amphisiella*, *Hemisincirra inquieta*, *Parasincirra* n. g., *Lamtostyla*, *Lamtostylides*, *Mucotrichidium* and *Paramphisiella*) or three (in *Terricirra* and *Spiroamphisiella*) anlagen;
5. Caudal cirri are formed in some taxa, i.e. *Spiroamphisiella*, *Paramphisiella* and *Mucotrichidium*, but not in others, i.e. *Amphisiella*, *Parasincirra* n. g., *Lamtostyla*, *Lamtostylides*, *Afroamphisiella*, *Hemisincirra inquieta* and *Terricirra*;
6. No transverse cirri are formed in *Afroamphisiella* and *Paramphisiella* whereas transverse cirri are formed in *Amphisiella*, *Parasincirra* n. g., *Lamtostyla*, *Lamtostylides*, *Terricirra*, *Mucotrichidium*, *Hemisincirra inquieta* and *Spiroamphisiella* [19, 21–23].

### Phylogenetic analyses

**Fig. 6:**
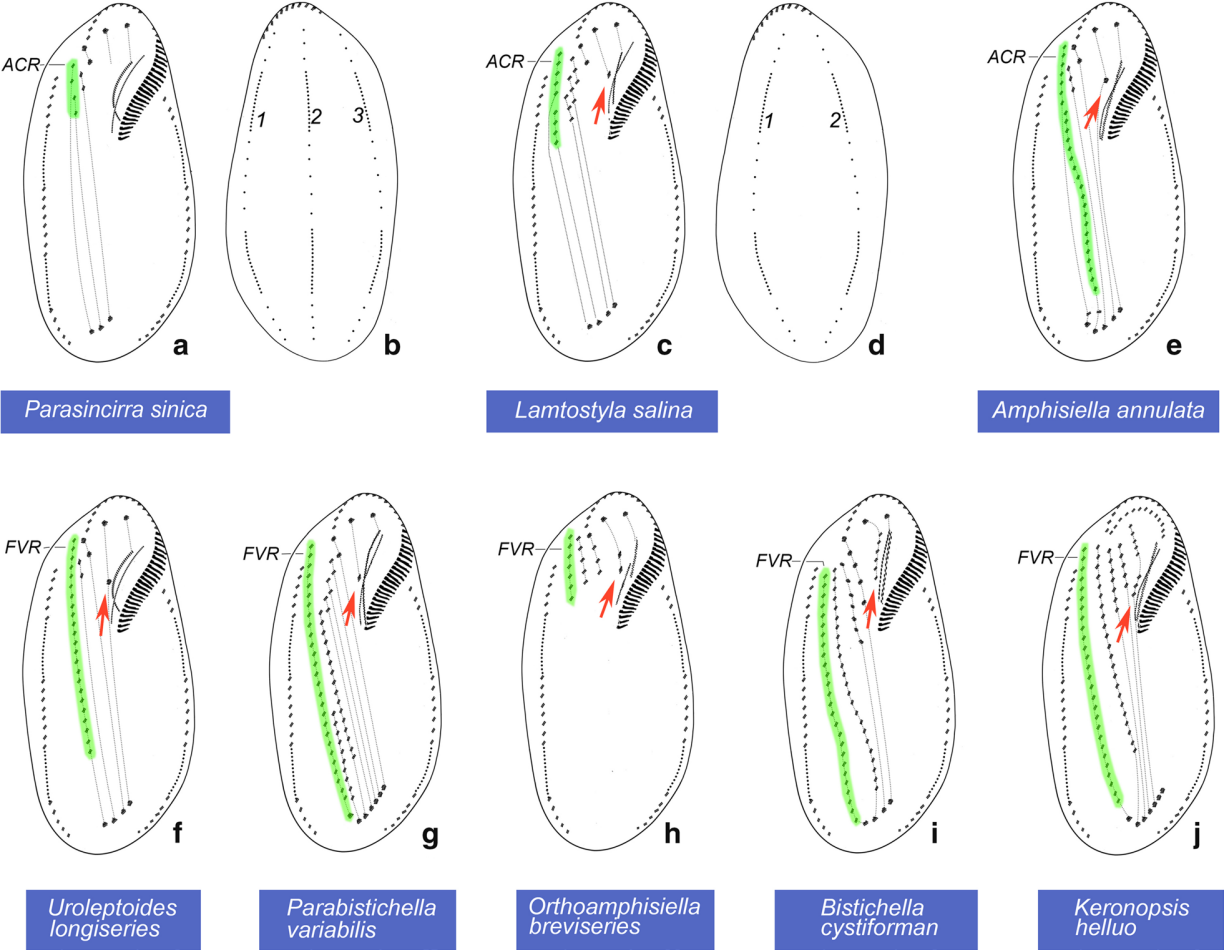
**a**–**j** Diagram of the infraciliature, and formation patterns of ventral cirri (dotted lines connecting cirri that develop from the same cirral streaks, arrows mark the buccal cirri) (a, c, e–j) and dorsal ciliature (b, d). **a** *Parasincirra sinica.* **b** *Parasincirra sinica, Amphisiella annulata, Uroleptoides longiseries, Parabistichella variabilis, Bistichella cystiformans* and *Keronopsis helluo*. **c** *Lamtostyla salina*. **d** *Lamtostyla salina* and *Orthoamphisiella breviseries.* **e** *Amphisiella annulata*. **f** *Uroleptoides longiseries*. **g** *Parabistichella variabilis*. **h** *Orthoamphisiella breviseries*. **i** *Bistichella cystiformans*. **j** *Keronopsis helluo* [19, 22, 24–26, 47, 48]

**Fig. 7:**
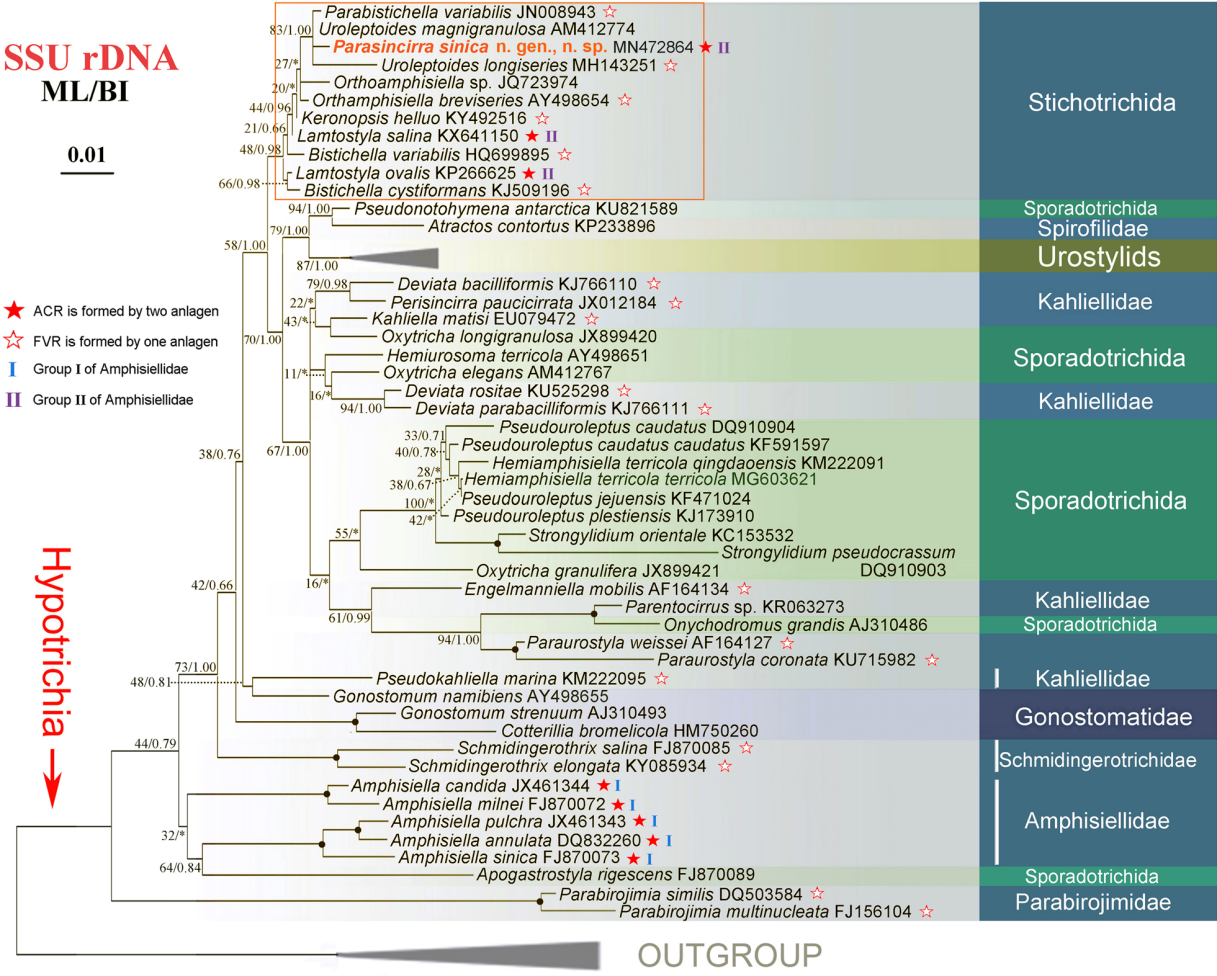
Maximum likelihood (ML) tree inferred from the SSU rDNA sequences showing the phylogenetic relationships of *Parasincirra sinica* n. sp. (in bold) and the related species (in rectangular box). Numbers near nodes are bootstrap values for maximum-likelihood and posterior probability values for Bayesian inference (BI). “*” at nodes indicates disagreement between the two methods. Fully supported (100%/1.00) branches are marked with solid circles. The scale bar corresponds to 0.01 expected substitutions per site

Molecular phylogenetic analyses did not resolve the relationship of the four polytomies represented by *Parasincirra sinica* n. sp*.*, *Uroleptoides magnigranulosa*, *U. longiseries* and *Parabistichella variabilis* (Figs. 6, 7). Taxonomically, *P. sinica* n. sp. has the critical character of the family Amphisiellidae, i.e., the ACR that originates from two separate anlagen, and apparently it should be assigned in this family (exactly, group II in Amphisiellidae) [18, 19]. However, the long ventral row is formed by just a single anlage in *Uroleptoides longiseries* and *Parabistichella variabilis,* hence they should not be assigned to Amphisiellidae. Whether *Uroleptoides magnigranulosa* is correctly assigned to the family Amphisiellidae needs further clarification [19, 24, 25]. The close relationship between these four species is supported by each having three enlarged frontal cirri, one marginal cirral row on each side and cortical granules present. Nevertheless, their close relationship not represented in the SSU rDNA tree might be due to poor taxon sampling.

The phylogenetic relationship between *Parasincirra sinica* n. sp. and its most morphologically similar genera, *Lamtostyla* and *Hemisinicirra*, also needs further investigation due to the remote position of *P. sinica* n. sp. in the SSU rDNA tree and the lack of information of the latter, e.g., the ontogenetic process has not been characterised for the type species of either.

## Conclusions

It is noteworthy that the positions of other amphisiellid species, even members of the type genus *Amphisiella*, are not well resolved well in the SSU rDNA tree (Fig. 7), which is consistent with previous studies [22, 26, 27]. Members of the Amphisiellidae are placed at two different parts of the phylogenetic tree even though they all possess the critical character of the family, i.e., the development of the ACR from two separate anlagen. Furthermore, the monophyly of the family Amphisiellidae is rejected by the AU test (p < 0.05) based on SSU-rDNA datasets. A composite row, resembling ACR, is formed in *Kahliella matisi* Vďačný et al., 2010, which belongs to the oxytrichine hypotrichs, and in *Hemiholosticha pantanalensis* Vďačný and Foissner, 2019, which belongs to the psilotrichid hypotrichs [28, 29]. These observations indicate that the ACR might be homoplastic. Further studies are needed to clarify the systematic position and evolution of species within the family Amphisiellidae.

## Methods

### Sample collection, isolation, and culturing

Sludge soil samples were collected from the upper 10 cm layer within a flood drain in Lanzhou (36º 3′ N; 103º 49′ E), China on 30 April 2017 (Fig. 1). Samples were dried at room temperature (about 24 °C) immediately after collection in order to preserve them. Several months later, ciliates were induced to excyst from the soil samples by employing the non-flooded Petri dish method [30]. Ciliate cells were then isolated using micropipettes and non-clonal cultures were established at room temperature in Petri dishes containing mineral water (Nongfu Spring) with rice grains added in order to stimulate the growth of bacteria as food source for the ciliates. We identified only one species, and relied on *in vivo* morphological characteristics to assure the accuracy of that identification for all downstream analyses, even though we were unable to establish clonal cultures. No other stichotrichid morphotypes were present in the protargol preparations.

### Morphology and morphogenetic studies

Live observations were carried out using bright field and differential interference contrast microscopy (Olympus BX53), photographed using a digital camera and figures were made by Photoshop. Protargol staining was used to reveal the ciliature and the nuclear apparatus [31]. The protargol reagent was synthesized following the protocol of Pan et al. (2013) [32]. Counts and measurements of stained specimens were performed at a magnification of 1000×. Drawings of protargol-stained cells were made with the assistance of a drawing device (camera lucida). To illustrate the changes that occurred during morphogenesis, parental structures are depicted by contour whereas new structures are shaded black [33, 34]. Terminology is according to Berger (2008) [19] and the systematic classification follows Lynn (2008) [18].

### DNA extraction, PCR amplification, and gene sequencing

Single cells of *Parasincirra sinica* n. sp. were isolated from cultures, washed several times with distilled water using a micropipette in order to remove potential contamination, and then transferred to 1.5 mL microfuge tubes with a minimum volume of water. DNA extraction was performed with the DNeasy Blood & Tissue Kit (Qiagen) following the manufacturer’s instructions with minor modifications [35, 36]. PCR amplification and sequencing of the SSU rDNA were performed according to Sheng et al. (2018) [37] using high fidelity Takara Ex Taq DNA polymerase (Takara Ex Taq; Takara Biomedicals) to minimise the possibility of amplification errors. The PCR products were purified using Geneclean (BIO 101 Inc., La Jolla, CA, USA) and sequenced bidirectionally on the ABI 3700 sequencer (GENEWIZ Biotechnology Co., Ltd., Beijing, China).

### Phylogenetic analyses and topology testing

The SSU rDNA sequence of the new species, together with 54 representative taxa downloaded from the GenBank database, were used in the phylogenetic analyses. The final alignment included 54 taxa and 1734 sites, with 446 variable sites and 265 parsimony-information sites. Three oligotrich species (*Novistrombidium sinicum* Liu et al., 2009, *Strombidium cuneiforme* Song et al., 2018 and *S. apolatum* Wilbert et al., 2005) were selected as putative outgroups. All sequences were aligned using the GUIDANCE web server (http://guidance.tau.ac.il/) [38]. The resulting alignment was manually edited using the program BioEdit 7.0 [39]. Both Maximum likelihood (ML) and Bayesian inference (BI) analyses were performed on the final alignment under the best-fit nucleotide substitution model of GTR + Γ that was selected by jModelTest ver. 2.1.7 [40]. The ML analysis was performed using RAxML-HPC2 on XSEDE v8.2.12 on the online server CIPRES Science Gateway [41], with 1000 rapid bootstrap replicates and a subsequent thorough ML search. Bayesian inference was computed with MrBayes on XSEDE 3.2.6 [42], running four Markov chains sampling every 100 generations for a million generations and discarding the first 25% of trees as burn-in. The majority rule consensus tree was produced from the remaining samples with each node labelled with its posterior probability. SeaView v.4 [43] and MEGA v5 [44] were used to visualise the tree topologies.

The approximately unbiased (AU) test [45] was performed to assess the monophyly of species of the family Amphisiellidae that possess an amphisiellid median cirral row. The constrained ML tree was generated based on SSU rDNA sequences. The site likelihoods for the resulting constrained topology and then on-constrained ML topology were calculated using PAUP and then analyzed in CONSEL [46].

## Data Availability

Sequence data are available in GenBank (Accession Numbers: MN472864 has been released). One protargol-stained slide (No. MJY2017043001B) with the holotype specimen and several paratype slides (No. MJY2017043001A, C) were deposited in the Laboratory of Protozoological Biodiversity and Evolution in Wetland, Shaanxi Normal University, China.

## Abbreviations

18S rDNA: Small subunit ribosomal DNA
ACR: Amphisiellid median cirral row
AZM: Adoral zone of membranelles
BI: Bayesian inference
bp: Base pairs
FVT-anlagen: Frontoventral-transverse cirral anlagen
GC: Guanine-cytosine
ML: Maximum likelihood
n. g.: Novum genus
n. sp.: Novum species

## References

[1] Chen L, Dong J, Wu W, Xin Y, Warren A, Ning Y (2020). Morphology and molecular phylogeny of a new hypotrich ciliate, *Anteholosticha songi* nov. spec., and an American population of *Holosticha pullaster* (Müller, 1773) Foissner et al., 1991 (Ciliophora, Hypotrichia). Eur J Protistol..

[2] Luo X, Huang JA, Li L, Song W, Bourland WA (2019). Phylogeny of the ciliate family Psilotrichidae (Protista, Ciliophora), a curious and poorly-known taxon, with notes on two algae-bearing psilotrichids from Guam, USA. BMC Evolut Biol.

[3] Kaur H, Negi SSRK, Kamra K (2019). Morphological and molecular characterization of *Neogastrostyla aqua* nov. gen., nov. spec. (Ciliophora, Hypotrichia) from River Yamuna, Delhi; comparison with *Gastrostyla*-like genera. Eur J Protistol..

[4] Park KM, Jung JH, Kim JH, Min GS, Kim S (2020). Morphology, morphogenesis, and molecular phylogeny of a new freshwater ciliate, *Gonostomum jangbogoensis* n. sp. (Ciliophora, Hypotricha), from Victoria Land, Antarctica. Eur J Protistol.

[5] Luo X, Yan Y, Shao C, Al-Farraj SA, Bourland WA, Song W (2018). Morphological, ontogenetic and molecular data support strongylidiids as being closely related to Dorsomarginalia (Protozoa, Ciliophora) and reactivation of the family Strongylidiidae Fauré-Fremiet, 1961. Zool J Linn Soc.

[6] Bharti D, Kumar S, La Terza A, Chandra K (2019). Morphology and ontogeny of *Tetmemena pustulata indica* nov. subspec. (Ciliophora, Hypotricha), from the Thane Creek, Mumbai, India. Eur J Protistol.

[7] Kim KS, Min GS (2019). Morphology and molecular phylogeny of *Oxytricha seokmoensis* sp. nov. (Hypotrichia: Oxytrichidae), with notes on its morphogenesis. Eur J Protistol..

[8] Xu W, Wang Y, Cheng T, Yu Y, El-Serehy H, Al-Farraj SA (2020). Reevaluation of the ‘well-known’ *Paraurostyla weissei* complex, with notes on the ontogenesis of a new *Paraurostyla* species (Ciliophora, Hypotrichia). Eur J Protistol.

[9] Dong J, Li L, Fan X, Ma H, Warren A (2020). Two *Urosoma* species (Ciliophora, Hypotrichia): a multidisciplinary approach provides new insights into their ultrastructure and systematics. Eur J Protistol.

[10] Wang Y, Jiang Y, Liu Y, Li Y, Katz LA, Gao F (2020). Comparative studies on the polymorphism and copy number variation of mtSSU rDNA in ciliates (Protista, Ciliophora): implications for phylogenetic, environmental, and ecological research. Microorganisms.

[11] Lian C, Luo X, Warren A, Zhao Y, Jiang J (2020). Morphology and phylogeny of four marine or brackish water spirotrich ciliates (Protozoa, Ciliophora) from China, with descriptions of two new species. Eur J Protistol.

[12] Jung JH, Berger H (2019). Monographic treatment of *Paraholosticha muscicola* (Ciliophora, Keronopsidae), including morphological and molecular biological characterization of a brackish water population from Korea. Eur J Protistol.

[13] Wang J, Zhao Y, Lu X, Lyu Z, Warren A, Shao C (2020). Does the *Gonostomum*-patterned oral apparatus in Hypotrichia carry a phylogenetic signal? Evidence from morphological and molecular data based on extended taxon sampling using three nuclear genes (Ciliophora, Spirotrichea). Sci China Life Sci..

[14] Zhang T, Dong J, Cheng T, Duan L, Shao C (2020). Reconsideration of the taxonomy of the marine ciliate *Neobakuella aenigmatica* Moon et al., 2019 (Protozoa, Ciliophora, Hypotrichia). Mar Life Sci Technol..

[15] Song W, Shao C (2017). Ontogenetic patterns of Hypotrich ciliates.

[16] Lyu Z, Wang J, Huang JA, Warren A, Shao C (2018). Multigene-based phylogeny of Urostylida (Ciliophora, Hypotrichia), with establishment of a novel family. Zool Scr.

[17] Fernandes NM, Schrago CG (2019). A multigene timescale and diversification dynamics of Ciliophora evolution. Mol Phylogenet Evol.

[18] Lynn DH (2008). The ciliated protozoa: characterization, classification and guide to the literature.

[19] Berger H (2008). Monograph of the Amphisiellidae and Trachelostylidae (Ciliophora, Hypotricha). Monogr Biol.

[20] Lu X, Huang JA, Shao C, Berger H (2018). Morphology, cell-division, and phylogeny of *Schmidingerothrix elongata* spec. nov. (Ciliophora, Hypotricha), and brief guide to hypotrichs with *Gonostomum*-like oral apparatus. Eur J Protistol..

[21] Chen X, Shao C, Lin X, Clamp JC, Song W (2013). Morphology and molecular phylogeny of two new brackish-water species of *Amphisiella* (Ciliophora, Hypotrichia), with notes on morphogenesis. Eur J Protistol.

[22] Dong J, Lu X, Shao C, Huang J, Al-Rasheid KAS (2016). Morphology, morphogenesis and molecular phylogeny of a novel saline soil ciliate, *Lamtostyla salina* n. sp. (Ciliophora, Hypotricha). Eur J Protistol..

[23] Li L, Zhao X, Ji D, Hu X, Al-Rasheid KAS, Al-Farraj SA (2016). Description of two marine amphisiellid ciliates, *Amphisiella milnei* (Kahl, 1932) Horváth, 1950 and *A. sinica* sp. nov. (Ciliophora: Hypotrichia), with notes on their ontogenesis and SSU rDNA-based phylogeny. Eur J Protistol..

[24] Wang J, Li J, Qi S, Warren A, Shao C (2019). Morphogenesis and molecular phylogeny of a soil cliate *Uroleptoides longiseries* (Foissner, Agatha and Berger, 2002) Berger 2008 (Ciliophora, Hypotrichia). J Eukaryot Microbiol.

[25] Jiang J, Huang J, Li L, Shao C, Al-Rasheid KAS, Al-Farraj SA (2013). Morphology, ontogeny, and molecular phylogeny of two novel bakuellid-like hypotrichs (Ciliophora: Hypotrichia), with establishment of two new genera. Eur J Protistol.

[26] Fan Y, Hu X, Gao F, Al-Farraj SA, Al-Rasheid KAS (2014). Morphology, ontogenetic features and SSU rRNA gene-based phylogeny of a soil ciliate, *Bistichella cystiformans* spec. nov. (Protista, Ciliophora, Stichotrichia). Int J Syst Evol Microbiol..

[27] Luo X, Yi Z, Gao F, Pan Y, Al-Farraj SA, Warren A (2017). Taxonomy and molecular phylogeny of two new brackish hypotrichous ciliates, with the establishment of a new genus (Protozoa, Ciliophora). Zool J Linn Soc.

[28] Vďačný P, Tirjaková E, Tóthová T, Pristaš P, Javorský P (2010). Morphological and phylogenetical studies on a new soil hypotrich ciliate: *Kahliella matisi* spec. nov. (Hypotrichia, Kahliellidae). Eur J Protistol..

[29] Vďačný P, Foissner W (2019). Morphology and ontogenesis of *Hemiholosticha pantanalensis* nov. spec. (Ciliophora, Hypotrichia, Psilotrichidae). Acta Protozool..

[30] Foissner W (2014). An update of ‘basic light and scanning electron microscopic methods for taxonomic studies of ciliated protozoa’. Int J Syst Evol Microbiol.

[31] Wilbert N (1975). Eine verbesserte Technik der Protargolimprägnation für Ciliaten. Mikrokosmos.

[32] Pan X, Bourland WA, Song W (2013). Protargol synthesis: an in-house protocol. J Eukaryot Microbiol.

[33] Wang J, Li J, Shao C (2020). Morphology, morphogenesis, and molecular phylogeny of a novel saline soil ciliate, *Heterourosomoida sinica* n. sp. (Ciliophora, Hypotrichia). Eur J Protistol..

[34] Shao C, Hu C, Fan Y, Warren A, Lin X (2019). Morphology, morphogenesis and molecular phylogeny of a freshwater ciliate, *Monomicrocaryon euglenivorum euglenivorum* (Ciliophora, Oxytrichidae). Eur J Protistol.

[35] Gao F, Gao S, Wang P, Katz LA, Song W (2014). Phylogenetic analyses of cyclidiids (Protista, Ciliophora, Scuticociliatia) based on multiple genes suggest their close relationship with thigmotrichids. Mol Phylogenet Evol.

[36] Wang Y, Wang C, Jiang Y, Katz LA, Gao F, Yan Y (2019). Further analyses of variation of ribosome DNA copy number and polymorphism in ciliates provide insights relevant to studies of both molecular ecology and phylogeny. Sci China Life Sci.

[37] Sheng Y, He M, Zhao F, Shao C, Miao M (2018). Phylogenetic relationship analyses of complicated class Spirotrichea based on transcriptomes from three diverse microbial eukaryotes: *Uroleptopsis citrina*, *Euplotes vannus* and *Protocruzia tuzeti*. Mol Phylogenet Evol.

[38] Penn O, Privman E, Ashkenazy H, Landan G, Graur D, Pupko T (2010). GUIDANCE: a web server for assessing alignment confidence scores. Nucleic Acids Res.

[39] Hall TA (1999). BioEdit: a user-friendly biological sequence alignment editor and analysis program for Windows 95/98/NT. Nucleic Acids Symp Ser.

[40] Darriba D, Taboada GL, Doallo R, Posada D (2012). JModelTest 2: more models, new heuristics and parallel computing. Nat Methods.

[41] Miller MA, Pfeiffer W, Schwartz T. Creating the CIPRES Science Gateway for inference of large phylogenetic trees. In: 2010 gateway computing environments workshop (GCE), New Orleans. 2010. p. 1–8.10.1109/GCE.2010.5676129.

[42] Ronquist F, Teslenko M, van der Mark P, Ayres DL, Darling A, Höhna S (2012). MrBayes 3.2: efficient Bayesian phylogenetic inference and model choice across a large model space. Syst Biol..

[43] Gouy M, Guindon S, Gascuel O (2010). SeaView version 4: a multiplatform graphical user interface for sequence alignment and phylogenetic tree building. Mol Biol Evol.

[44] Tamura K, Peterson D, Peterson N, Stecher G, Nei M, Kumar S (2011). MEGA5: molecular evolutionary genetics analysis using maximum likelihood, evolutionary distance, and maximum parsimony methods. Mol Biol Evol.

[45] Shimodaira H (2002). An approximately unbiased test of phylogenetic tree selection. Syst Biol.

[46] Shimodaira H, Hasegawa M (2001). Consel: for assessing the confidence of phylogenetic tree selection. Bioinformatics.

[47] Park KM, Chae N, Jung JH, Min GS, Kim S, Berger H (2017). Redescription of *Keronopsis helluo* Penard, 1922 from Antarctica and *Paraholosticha pannonica* Gellért and Tamás, 1959 from Alaska (Ciliophora, Hypotricha). Eur J Protistol.

[48] Berger H (2011). Monograph of the Gonostomatidae and Kahliellidae (Ciliophora, Hypotricha). Monogr Biol.

